# Inhouse Bridging Thrombolysis Is Associated With Improved Functional Outcome in Patients With Large Vessel Occlusion Stroke: Findings From the German Stroke Registry

**DOI:** 10.3389/fneur.2021.649108

**Published:** 2021-06-10

**Authors:** Ilko L. Maier, Andreas Leha, Mostafa Badr, Ibrahim Allam, Mathias Bähr, Ala Jamous, Amelie Hesse, Marios-Nikos Psychogios, Daniel Behme, Jan Liman

**Affiliations:** ^1^Department of Neurology, University Medical Center Göttingen, Göttingen, Germany; ^2^Department of Medical Statistics, University Medical Center Göttingen, Göttingen, Germany; ^3^Department of Neuroradiology, University Medical Center Göttingen, Göttingen, Germany; ^4^Department of Neuroradiology, Universitätsspital Basel, Basel, Switzerland; ^5^Department of Neuroradiology, University Hospital Magdeburg, Magdeburg, Germany

**Keywords:** ischemic stroke, LVOS, bridging, endovascular treatment, rtPA

## Abstract

**Background:** Endovascular treatment (EVT) for large vessel occlusion stroke (LVOS) is highly effective. To date, it remains controversial if intravenous thrombolysis (IVT) prior to EVT is superior compared with EVT alone. The aim of our study was to specifically address the question, whether bridging IVT directly prior to EVT has additional positive effects on reperfusion times, successful reperfusion, and functional outcomes compared with EVT alone.

**Methods:** Patients with LVOS in the anterior circulation eligible for EVT with and without prior IVT and direct admission to endovascular centers (mothership) were included in this multicentric, retrospective study. Patient data was derived from the German Stroke Registry (an open, multicenter, and prospective observational study). Outcome parameters included groin-to-reperfusion time, successful reperfusion [defined as a Thrombolysis in Cerebral Infarction (TICI) scale 2b-3], change in National Institute of Health Stroke Scale (NIHSS), modified Rankin Scale (mRS), and mortality at 90 days.

**Results:** Of the 881 included mothership patients with anterior circulation LVOS, 486 (55.2%) received bridging therapy with i.v.-rtPA prior to EVT, and 395 (44.8%) received EVT alone. Adjusted, multivariate linear mixed effect models revealed no difference in groin-to-reperfusion time between the groups (48 ± 36 vs. 49 ± 34 min; *p* = 0.299). Rates of successful reperfusion (TICI ≥ 2b) were higher in patients with bridging IVT (fixed effects estimate 0.410, 95% CI, 0.070; 0.750, p = 0.018). There was a trend toward a higher improvement in the NIHSS during hospitalization [ΔNIHSS: bridging-IVT group 8 (IQR, 9.8) vs. 4 (IQR 11) points in the EVT alone group; fixed effects estimate 1.370, 95% CI, −0.490; 3.240, *p* = 0.149]. mRS at 90 days follow-up was lower in the bridging IVT group [3 (IQR, 4) vs. 4 (IQR, 4); fixed effects estimate −0.350, 95% CI, −0.680; −0.010, *p* = 0.041]. There was a non-significantly lower 90 day mortality in the bridging IVT group compared with the EVT alone group (22.4% vs. 33.6%; fixed effects estimate 0.980, 95% CI −0.610; 2.580, *p* = 0.351). Rates of any intracerebral hemorrhage did not differ between both groups (4.1% vs. 3.8%, *p* = 0.864).

**Conclusions:** This study provides evidence that bridging IVT might improve rates of successful reperfusion and long-term functional outcome in mothership patients with anterior circulation LVOS eligible for EVT.

## Introduction

Endovascular treatment (EVT) of large vessel occlusion stroke (LVOS) has been shown to be highly effective and superior to intravenous thrombolysis (IVT) alone in multiple studies (1–3). However, the role of bridging therapy with IVT prior to EVT still is a matter of debate with studies showing no additional effect of IVT (4–6) and studies showing beneficial effects on functional outcome and reperfusion rates (7–9). Within the HERMES trials, most patients received IVT prior to endovascular treatment (MrClean 87%; ESCAPE 72%) (1). The conclusion of these trails to date is that IVT prior to thrombectomy is safe and still should be the standard of care. Limitations of these studies are the differences in study design, a lack of “real world” data with highly selected patient groups, the inclusion of heterogeneous patient groups (mothership, drip and ship, and just ship), and the fact that thrombectomy techniques as well as symptom to reperfusion times significantly improved in the last few years. Moreover, the patient numbers of most studies addressing the effect of bridging thrombolysis are low, and most meta-analysis/analysis from registries included patient data from the pre-HERMES studies era and do not differentiate between patients with drip-and-ship IVT and patients receiving IVT directly prior to EVT (“mothership” patients).

To address the role of these limitations and to clarify the role of IVT prior to EVT, the large, well-designed prospective DIRECT-MT study, including 656 patients enrolled at 41 academic tertiary care centers in China, recently demonstrated non-inferiority of the direct-EVT compared with the bridging-IVT approach with regard to 90 days functional outcome, despite a higher rate of successful reperfusions in the bridging IVT group (10). Why in this study, a higher reperfusion state prior to EVT, and a higher reperfusion rate achieved by EVT in combination with IVT, did not lead to improved functional outcomes in the bridging group, is not entirely clear and still a matter of debate. However, the DIRECT-MT trial had some shortcomings, which need to be considered for the interpretation of the results: First, the median door to needle time in this trial was 59 min. Given the fact that especially the effect of rtPa is highly time dependent, and that goal door to needle times in Europe are in the range of 30 min, the possible effect of rtPa might have been underestimated in this trial, although a higher percentage of successful reperfusions before EVT was observed in the trial. Second, as some patients had to pay for the rtPa treatment, this might even further have influenced the time scale of the iv treatment. Finally, there was a significant difference in patients not undergoing EVT between IVT + EVT vs. the EVT alone group. These facts might explain, why—despite reporting a successful reperfusion rate of >80%—the percentage of patients with favorable functional outcome with 36.6% was lower compared with previous pooled analyses of large thrombectomy trials with 46% favorable functional outcome (1, 10). The differences in functional outcome between the Chinese DIRECT-MT trial and the previous, large western thrombectomy trials are likely to be multifactorial including the difference in the studied ethnical group (Asian vs. Caucasian population with different stroke etiologies and subtypes).

Therefore, the aim of our study was to specifically address the role of in-house (mothership) bridging IVT directly prior to mechanical thrombectomy and to compare reperfusion times and reperfusion rates as well as functional outcome and complications in patients with and without bridging IVT prior to EVT.

## Materials and Methods

### Patient Population and Clinical Characteristics

Available data of patients enrolled in the German Stroke Registry—Endovascular Treatment (GSR-ET 07/2015-04/2018; ClinicalTrials.gov Identifier: NCT03356392) between 2016 and 2019 was analyzed. The GSR-ET is an ongoing, open-label, prospective, multicenter registry of 25 sites in Germany, collecting consecutive patients with LVOS undergoing EVT. This registry includes neuroradiological and neurological data as well as all time metrics relevant to the interventional treatment and clinical outcome of patients presenting with LVOS. In detail, time metrics and imaging characteristics were recorded by a stroke-experienced senior neuroradiologist, while clinical data like prior medical history and medication, National Institute of Health Stroke Scale (NIHSS), and modified Rankin scale (mRS) have been evaluated and recorded by an experienced, stroke-trained neurologist. NIHSS was recorded at initial presentation of the patient in the emergency department and at discharge. mRS was recorded at discharge and at 90 days follow-up. The endovascular approaches (direct aspiration, stent retrieval, i.e., thrombolysis and combinations of these approaches) were based on the judgment of the treating neuroradiologist. For further information and main outcome of the GSR, we refer to the original publication of the main outcome (11).

### Treatment Groups

We predefined two treatment groups: the first treatment group received IVT directly prior the EVT within a time window of <4.5 h and after exclusion of contraindications according to the American Heart Association (AHA)–American Stroke Association (ASA) guidelines (12). In this group, for the thrombolytic therapy only, Alteplase was used and administered right after the native CT-scan if intracerebral hemorrhage had been ruled out (0.9 mg/kg over 1 h with 10% of initial bolus).The second treatment group received EVT alone. Both treatment groups were directly admitted at a thrombectomy center and had not been transferred from another hospital (“mothership” patients). Only patients with anterior circulation LVOS (occlusions of the extra- or intracranial carotid artery or occlusions of the medial cerebral artery in its M1 and M2 segment) were included in the analysis. We excluded patients being inconsistently recorded or had missing data (both regarding IVT treatment and time metrics), non-mothership cases, patients with other occlusions than ICA and MCA occlusions, flow restoration with IVT only prior to EVT, and patients with incomplete IVT treatment independent of the reason.

### Outcome Measures

We defined functional (peri-)procedural and safety measures as follows: mRS and mortality at 90 days, change of NIHSS from admission to discharge (ΔNIHSS = NIHSS at admission minus NIHSS at discharge), groin to reperfusion times [time from groin puncture to first angiographic series with Thrombolysis in Cerebral Infarction perfusion scale (TICI) ≥ 2b], rates of successful recanalization (defined as TICI ≥ 2b) as well as any intracerebral hemorrhage, groin hematoma, groin pseudoaneurysm, space occupying edema of medial cerebral artery territory, myocardial infarction, and recurrent stroke.

### Statistical Analysis

All variables are summarized by either mean ± SD, median with interquartile range (IQR) or absolute and relative frequencies, as appropriate. Values were compared univariately between the groups using Welch's two-sample *t*-test, Fisher's exact test, or Mann–Whitney U test as appropriate. Linear mixed effect models taking into account the center as random effect, and controlling for the following potential confounders unequally distributed in a univariate analysis with a *p* < 0.2: Onset-to-first TICI ≥ 2b- and onset-to-imaging times, diabetes mellitus, arterial hypertension, atrial fibrillation, premedication with acetylsalicylic acid, clopidogrel, low molecular weight heparin, oral anticoagulants (Apixaban, Rivaroxaban, Dabigatran, Edoxaban, and Marcumar), living status, pre-stroke-modified Rankin score (mRS) and kind of sedation as well as intracranial internal carotid artery bifurcation occlusion, and Alberta stroke program early CT score. These confounders were fit to the data to assess the association between IVT-treatment and the groin-to-reperfusion status and time as well as the functional outcomes. The scores of the mRS were modeled using mixed effect ordinal (cumulative link) regression models (13). Missing values were imputed using multiple imputations.

The 3-month mortality was modeled using a mixed effect logistic regression model. In order to assess a potential power limitation in the fully controlled model, as a sensitivity analysis, a propensity score analysis was performed: logistic mixed effect regression model was fit to the grouping using the potential confounders as fixed effects (and the center as random effect) and the fitted logit scores were used as propensity scores, which were added to the model for the 3-month mortality as covariable. Additionally, we performed an analysis on 1:1 matched samples where samples were matched within centers using a caliper of 0.15. Data from propensity score matched samples were used to plan for a comparison using the Mann–Whitney U-test in a future randomized trial. For the mRS at 3 months, we conducted two power analyses to detect differences between EVT + IVT and EVT – IVT: In the first scenario, the power to detect the observed difference was analyzed. The second scenario assumes a smaller effect of 20% of the patients receiving a smaller mRS in the EVT + IVT group. The significance level was set to alpha = 5% for all statistical tests. All analyses were performed with the statistic software R using the R-package lme4 (14) for the mixed effect logistic regression, the R-package ordinal (15) for the mixed effect cumulative link models, the R-package CMatching (16) for the clustered propensity matching, and the R-package WMWssp (17) for the power analyses for the Mann–Whitney U-test.

## Results

### Baseline Characteristics

At the time of data analysis, the GSR databank contained 2,637 cases. After discarding cases being inconsistently recorded, cases with missing data, non-mothership cases, and patients with other occlusions than ICA and MCA occlusions, 881 patients remained for the analysis (Supplementary Figure 1). From these patients, 486 (55.2%) received bridging-IVT prior to EVT, and 395 (44.8%) received EVT alone. Baseline characteristics of both groups are shown in Table 1.

**Table 1:** Baseline characteristics of with (EVT + IVT) and without (EVT – IVT) bridging thrombolysis.

	**EVT + IVT group!!break (*n* = 486)**	**EVT – IVT!!break (*n* = 395)**	***p*-value**
**Demographics and clinical data**			
Age (mean ± SD)	72 ± 13	73 ± 13	0.330
Sex male (*n*, %)	237 (48.9)	190 (48.1)	0.839
Baseline NIHSS (median score, IQR)	14 (10,18)	15 (10,19)	0.279
Pre-stroke mRS (median score, IQR)	0 (0;1)	0 (0;2)	<0.001
Living status			0.038
Home (*n*, %)	403 (87.0%)	313 (83.7%)	
Nursing at home (*n*, %)	17 (3.7%)	29 (7.8%)	
Nursing home (*n*, %)	43 (9.3%)	32 (8.6%)	
Stroke etiology			<0.001
Cardioembolism (*n*, %)	232 (48.2%)	209 (53.7%)	
Large-artery arteriosclerosis (*n*, %)	125 (26.0%)	91 (23.4%)	
Other determined etiology (*n*, %)	17 (3.5%)	29 (7.5%)	
Undetermined etiology (*n*, %)	90 (18.7%)	57 (14.7%)	
Anesthesia			0.009
CS (*n*, %)	114 (24.4%)	109 (28.6%)	
Switch from CS to GA (*n*, %)	26 (5.6%)	7 (1.8%)	
GA (*n*, %)	327 (70.0%)	265 (69.6%)	
**Medical history**			
Diabetes mellitus (*n*, %)	93 (19.2%)	95 (24.1%)	0.083
Arterial hypertension (*n*, %)	349 (72.1%)	308 (78.0%)	0.051
History of AF (*n*, %)	176 (36.5%)	179 (45.5%)	0.007
Dyslipoproteinemia (*n*, %)	165 (34.2%)	151 (38.4%)	0.203
Previous and current smoking (*n*, %)	107 (22%)	95 (24%)	0.629
**Time metrics**			
Onset-to-needle time (mean min ± SD)	101 ± 54	n.a.	n.a.
Onset-to-imaging time (mean min ± SD)	84 ± 53	103 ± 163	0.177
Onset-to-groin time (mean min ± SD)	159 ± 66	168 ± 84	0.281
Onset-to-first TICI≥2b (mean min ± SD)	205 ± 76	217 ± 82	0.146
**Imaging data**			
ASPECTS at baseline (median, IQR)	9 (8,10)	8 (7,10)	<0.001
**Site of vessel occlusion**			
Intracranial ICA bifurcation (*n*, %)	75 (15.4%)	82 (20.8%)	0.042
Intracranial ICA non-bifurcation (*n*, %)	22 (4.5%)	22 (5.6%)	0.535
Extracranial ICA (*n*, %)	17 (3.5%)	20 (5.1%)	0.311
MCA, proximal M1-segent (*n*, %)	201 (41.4%)	164 (41.5%)	1.000
MCA, distal M1-segent (*n*, %)	121 (24.9%)	85 (21.5%)	0.263
MCA, M2-segent (*n*, %)	111 (22.8%)	81 (20.5%)	0.413
**Medication on admission**			
Acetylsalicylic acid (*n*, %)	117 (30.4%)	185 (39.4%)	0.006
Clopidogrel (*n*, %)	8 (1.7%)	18 (4.7%)	0.015
Low molecular weight heparin (*n*, %)	2 (0.4%)	18 (4.7%)	<0.001
Apixaban (*n*, %)	2 (0.4%)	30 (7.8%)	<0.001
Rivaroxaban (*n*, %)	4 (0.9%)	24 (6.2%)	<0.001
Dabigatran (*n*, %)	1 (0.2%)	5 (1.3%)	0.096
Edoxaban (*n*, %)	2 (0.4%)	12 (3.1%)	0.002
Marcumar (*n*, %)	14 (3.0%)	38 (9.9%)	<0.001

*EVT, endovascular therapy; IVT, intravenous thrombolysis; SD, standard deviation; IQR, interquartile range; NIHSS, National Institute of Health Stroke Scale; mRS, modified Rankin Scale; CS, conscious sedation; GA, general anesthesia; AF, atrial fibrillation; TICI, thrombolysis in cerebral infarction scale; ASPECTS, Alberta stroke programme early CT score; ICA, internal carotid artery; MCA, medial cerebral artery*.

Patients with bridging-IVT had significantly lower pre-stroke mRS (*p* < 0.001) and were less likely to have cardiovascular comorbidities like diabetes mellitus (19.2% vs. 24.1%, *p* = 0.083), arterial hypertension (72.1% vs. 78%, *p* = 0.051), and atrial fibrillation (36.5% vs. 45.5%, *p* = 0.007) as well as were significantly less likely to be on antiplatelets or anticoagulants. In addition, patients with bridging-IVT were more likely to live at home without nursing, while the percentage of patients living in a nursing home was equally distributed. There was no significant difference in symptom onset to imaging—(84 ± 53 min vs. 103 ± 163 min, *p* = 0.177), groin- (159 ± 66 min vs. 168 ± 84 min, *p* = 0.281) and to reperfusion times (205 ± 76 min vs. 217 ± 82 min, *p* = 0.146). Patients with bridging IVT had lower rates of occlusions of the intracranial internal carotid artery bifurcation (15.4% vs. 20.8%, *p* = 0.042); all other sites of vessel occlusion were equally distributed. Alberta stroke programme early CT score (ASPECTS) was higher in the bridging group (9 vs. 8, *p* < 0.001); there were no differences in adverse events between both groups, including any kind of intracerebral hemorrhage (Supplementary Table 1).

### Unadjusted Analysis of Primary Outcomes

In the unadjusted analysis of the outcome parameters, there was no difference between groin to reperfusion times and reperfusion status on final angiogram between the bridging- and EVT alone group (Table 2). Patients with bridging-IVT had lower NIHSS at discharge (4 vs. 7, *p* < 0.001), higher improvement on the NIHSS during in-patient stay (8 vs. 4, *p* = 0.001) as well as lower mRS at discharge and at 90 days follow-up (3 vs. 4, *p* < 0.001). Mortality rates in the bridging-IVT group were lower compared with the EVT alone group [96 (22.5%) vs. 119 (33.6%), *p* = 0.001)].

**Table 2:** Unadjusted primary outcome parameter in patients with and without bridging-thrombolysis.

	**EVT + IVT group (*n* = 486)**	**EVT – IVT group (*n* = 395)**	***p*-value**
**Procedural and imaging outcomes**			
Groin-to-first TICI≥2b	48 ± 36	49 ± 34	0.766
Final TICI-score			0.612
TICI 0	50 (5.7%)	27 (6.9%)	
TICI 1	12 (1.4%)	6 (1.5%)	
TICI 2a	31 (6.4%)	29 (7.4%)	
TICI 2b	187 (38.7%)	140 (35.8%)	
TICI 3	236 (48.9%)	189 (48.3%)	
**Functional outcomes**			
NIHSS discharge (median points, IQR)	4 (9)	7 (12)	<0.001
Δ NIHSS (median points, IQR)	8 (9.8)	4 (11)	0.001
mRS at discharge (median score, IQR)	3 (4)	4 (3)	<0.001
mRS after 90 days (median score, IQR)	3 (4)	4 (4)	<0.001
Mortality at 90 days (*n*, %)	96 (22.5%)	119 (33.6%)	0.001

*EVT, endovascular treatment; IVT, intravenous thrombolysis; TICI, thrombolysis in cerebral infarction scale, IQR, interquartile range; NIHSS, National Institute of Health Stroke Scale; mRS, modified Rankin Scale; Δ NIHSS = NIHSS admission minus NIHSS discharge*.

### Adjusted Analysis of Primary Outcomes

After adjustment for multiple confounders, successful reperfusion (defined as TICI ≥ 2b on final angiogram) was associated with bridging-IVT (fixed effects estimate 0.410, 95% CI, 0.070; 0.750, *p* = 0.018), while no difference persisted with regard to groin to reperfusion times between both groups (fixed effects estimate −0.030, 95% CI, −0.070; 0.020, *p* = 0.243) (Table 3). Concerning the adjusted analysis of the functional outcome parameters, bridging IVT was associated with lower mRS at discharge (fixed effects estimate −0.340, 95% CI, −0.650; −0.030, *p* = 0.031) and at 90 days follow-up (fixed effects estimate −0.350, 95% CI, −0.680; −0.010, *p* = 0.041).

**Table 3:** Adjusted analysis of outcome parameters and bridging IVT using linear mixed effect models.

	***n***	**Fixed effects estimate**	**95% CI**	***p*-value**
Successful reperfusion	704	0.410	0.070; 0.750	0.018
Groin-to-reperfusion time	604	−0.030	−0.070; 0.020	0.243
mRS at discharge	693	−0.340	−0.650; −0.030	0.031
mRS at 90 days	622	−0.350	−0.680; −0.010	0.041
NIHSS at discharge	554	−0.050	−0.130; 0.030	0.209
Δ NIHSS	552	1.370	−0.490; 3.240	0.149
Mortality	622	0.980	−0.610; 2.580	0.346

*IVT, intravenous thrombolysis; CI, confidence interval; mRS, modified Rankin scale; NIHSS, National Institute of Health Stroke Scale; Δ NIHSS, NIHSS admission minus NIHSS discharge, successful reperfusion, TICI, thrombolysis in cerebral infarction scale ≥ 2b on final angiogram*.

In addition, patients with bridging-IVT had lower NIHSS at discharge (fixed effects estimate −0.050, 95%CI, −0.130; 0.030, *p* = 0.209) and higher improvement in NIHSS between admission and discharge (Δ NIHSS; fixed effects estimate 1.370, 95% CI, −0.490; 3.240, *p* = 0.149), in which both did not reach statistical significance after correcting for multiple confounders (Table 3). Adjusted mortality rates were non-significantly lower in the bridging IVT group (fixed effects estimate 0.980, 95% CI −0.610; 2.580, *p* = 0.351). Also, in the propensity score adjusted model, no significant group effect was observed (estimate 0.770, 95% CI 0.451; 1.315, *p* = 0.338). Similarly, in the propensity score matched set, no significant group effect was observed (estimate 0.833, 95% CI 0.534–1.297, *p* = 0.418). An overview and visualization of all model covariates is given in Supplementary Figures 2A–E.

## Discussion

In this study, we found an association between bridging IVT and higher rates of successful reperfusion as well as improved functional outcome including a “real world” cohort of patients receiving in-house bridging-IVT vs. EVT alone for anterior circulation LVOS in multiple tertiary stroke centers in Germany.

The treatment approach of bridging-IVT has been suspected to exhibit multiple potential advantages compared with EVT alone. These advantages include earlier and more complete reperfusion, especially in delayed intervention and if the thrombus is challenging to reach, dissolution of distal thrombus fragments by IVT as well as reperfusion of the vessel before initiation of the interventional procedure. In contrast, possible delays of EVT, risks for intracerebral hemorrhage, and increased costs have to be taken into account (18). Most retrospective studies and *post hoc* analyses from randomized controlled clinical trials on the question if bridging with IVT is necessary prior to EVT have found benefits compared with EVT alone (19, 20). As all these studies—including the present study—have major limitations inherent to retrospective study designs, the large, multi-center and prospective DIRECT-MT trial has recently been conducted in China. Interestingly, this study also found a higher percentage of successful reperfusion in patients with the combined treatment with IVT and EVT (our study: 87% vs. 83%; DIRECT-MT: 85% vs. 79%), while the groin to reperfusion times (our study: 48 min vs. 49 min; DIRECT-MT: 71 min vs. 60 min) as well as the incidence of brain hemorrhage did not differ significantly between the groups (10). The overall lower reperfusion rate and longer groin to reperfusion time of the DIRECT-MT study compared with our data could be discussed as reasons for a lack of effect on functional outcome (21). Moreover, from a statistical point of view, the margin for non-inferiority in the DIRECT-MT study was generous and the confidence intervals did not exclude a benefit of ~20% in the group treated with IVT. Recently, the Japanese trial (SKIP study) was published, which also did not show inferiority in the EVT only group (22). However, this trial also showed numerically more patients achieving good reperfusion (>TICI2b) as well as excellent outcome (mRS 0–1) in the IVT + EVT group. Both secondary endpoints were not statistically significant. First, the reason for this could be because of the lower rtPA dose (0.6 mg/kg), which is used in Japan, second, because of the total small sample size (n = 100 in each group), which was originally calculated using the results from trials using 0.9 mg/kg of Alteplase (22). This point is also discussed as a limitation by the authors themselves. Another widely discussed shortcoming of the two abovementioned studies is the raw segmentation of the mRS scheme itself, especially when it comes to smaller, but clinically highly relevant add-on effects like cognitive endpoints (23). We performed a power analysis based on our data for the day 90 mRS with a strictly propensity score matched sample (*n* = 332) for a comparison using the Mann–Whitney U-test. To detect a difference as pronounced as in the data, 350 subjects would suffice. If the effect is smaller, of course, more subjects are necessary: if 20% of the samples end up with a smaller mRS at day 90 with IVT, 2,336 subjects would be necessary. Currently, there are three more trials ongoing [MR CLEAN-NO IV (ISRCTN80619088), SWIFT DIRECT (NCT03192332), and DIRECT-SAFE (NCT03494920)], which are necessary to give more solid information. Additionally, these trials could help to perform a meta-analysis in order to provide more clarity.

The reason why groin to reperfusion times were not shorter in the bridging IVT group, but the rate of successful reperfusion was higher, seems to be contradictory. One would assume that IVT facilitates the clot removal by reducing clot load and softening the thrombus and therefore improving the passage through the thrombus and its removal. The lack of difference between groin to reperfusion times in our study could be explained by the different stroke etiologies in both groups. Most importantly, there were around 5% more cardio-embolic strokes and 2.6% less macroangiopathic strokes in the EVT alone group. While IVT might have facilitating effects in both stroke etiologies, cardio-embolic thrombi removal by EVT in the majority of cases is faster and easier compared with often hard, calcified, and plaque-associated thrombi and emboli (24, 25). One could assume equal effects of IVT in both groups, while time to reperfusion has been shorter in the EVT alone group because of technically easier clot removals in this group requiring less passes and aspirations. The higher rate of successful reperfusion is mechanistically plausible, as rates of reocclusions and residual thrombi are likely to be reduced by IVT after clot removal and the assumption, that proximal parts of the thrombi are being dissolved (reduction of thrombus length) and possibly emboli in new territories could be resolved, though, due to the study design, we were not able to analyze the original CTA-scans for this purpose, and no data is available if and when follow-up CTA scans were performed.

Multiple effects of IVT improving functional outcomes have been discussed. IVT prior to EVT could lead to the lysis of small, peripheral thrombi impairing the penumbra-perfusion by collaterals. As the majority of large vessel occlusions are likely to be of embolic origin, the occlusion of collaterals by shattered thrombi therefore might be crucial for the functional outcome. Consequently, it could be speculated that the collateralization could have been positively influenced by the systemic administration of IVT, which has been administered after CTA, while this effect was missing in the non-bridging-group. In addition, also sources of emboli like cardiac thrombi are being treated by IVT, and the rate of recurrent strokes could be lower in this group. In this respect, Molina et al. showed that M1 occlusions of cardioembolic source are more likely to be recanalized by IVT compared with other sources of thrombus origin (26).

### Strengths and Limitations

Limitations of previous retrospective studies on the role of bridging IVT include monocentric designs, lack of sufficient patient and periprocedural data with potential bias, and the inclusion of heterogeneous patient groups (e.g., inclusion of drip-and-ship patients and non-anterior circulation occlusions) (27). In contrast, the strength of our study is the inclusion of a large cohort of highly selected patients being treated in multiple German thrombectomy centers, receiving full doses of IVT directly prior to EVT for anterior circulation LVOS. Although we adjusted our regression analyses for multiple confounders like comorbidities, pre-stroke medication, peri-interventional factors including time metrics and kind of anesthesia, stroke severity, and pre-stroke functional status, residual confounding is still possible. The most important bias in this respect is represented by the various reasons not to treat with bridging-IVT (selection bias), which were at the discretion of the treating neurologists and neuroradiologist using different clinical (e.g., age of the patient) and imaging-based (e.g., cerebral microangiopathy) factors. Contraindications for IVT include cancer, recent surgery, and current anticoagulation. The first two factors can be major contributing factors for worse functional outcomes, for which in this study no correction could be made, as these data were not recorded in the GSR. However, from a clinical point of view, these patients represent a minority of EVT patients, and therefore, this bias seems to be negligible. In contrast, anticoagulation is highly associated with existing atrial fibrillation, which again is more prevalent in patients with a high number of comorbidities. On the one hand, residual bias concerning other comorbidities significantly influencing the functional outcome of the possibly higher morbidity of patients in the sole EVT group cannot be entirely excluded. On the other hand, in the GSR, only any kind of intracerebral hemorrhage in the post-interventional phase is recorded, not differentiating symptomatic from asymptomatic hemorrhages. This again represents another limitation of our study and should be considered when interpreting the results, even if total rates of intracerebral hemorrhages did not differ between both groups. In addition, the aim of the study was to investigate only patients with and without bridging IVT actually undergoing EVT. Therefore, an additional effect of IVT-related racialization without EVT on functional outcome is possible. Finally, 316 cases have been excluded because of inconsistent or missing data regarding IVT treatment times and additional 38 cases were excluded because of recanalization after IVT prior to EVT. Concerning this significant number of excluded patients, additional selection bias is possible.

In conclusion, our findings provide further evidence for the effectiveness and safety of bridging IVT directly prior to EVT, with all precautions due to the retrospective design. Thus, the findings of the ongoing prospective, randomized trials are highly anticipated and will hopefully finally answer the question, if and for which kind of patient bridging IVT is necessary and in which scenarios is dispensable.

## Data Availability Statement

The raw data supporting the conclusions of this article will be made available by the authors, without undue reservation.

## Ethics Statement

The studies involving human participants were reviewed and approved by Ethics commission of the university medicine Göttingen; 16/2/16. The patients/participants provided their written informed consent to participate in this study.

## Author Contributions

IM designed the study, was involved in the acquisition and statistical analysis of the data, drafted and finalized the manuscript, and approved the manuscript before submission. AL was involved in the statistical analysis of the data and approved the manuscript before submission. MBad, IA, AH, and were involved in the acquisition of the data and drafting of the manuscript and approved the manuscript before submission. MBäh contributed to the manuscript and approved the manuscript before submission. DB contributed to the manuscript, involved in the acquisition of the data, and approved the manuscript before submission. M-NP contributed to the manuscript, involved in the acquisition of the data, and approved the manuscript before submission. JL contributed to the manuscript and approved the manuscript before submission. Patient data were collected by the GSR-ET committee. All authors contributed to the article and approved the submitted version.

## Conflict of Interest

M-NP received speakers' honoraria from Siemens Healthineers. The remaining authors declare that the research was conducted in the absence of any commercial or financial relationships that could be construed as a potential conflict of interest. The handling Editor declared a shared affiliation, though no other collaboration, with one of the authors, M-NP. The reviewer FF declared a past co-authorship with one of the authors M-NP to the handling Editor.
