# Supplementary material for: Inhouse Bridging Thrombolysis Is Associated With Improved Functional Outcome in Patients With Large Vessel Occlusion Stroke: Findings From the German Stroke Registry
Source: Front Neurol. 2021 Jun 10;12:649108. doi: 10.3389/fneur.2021.649108 (PMC8222775; doi:10.3389/fneur.2021.649108)
Supplement: Supplementary file 1 [file Table_1.DOCX]

| Supplementary Table 1: Adverse events in patients with (EVT+IVT)- and without (EVT-IVT) bridging thrombolysis | | | | |
| --- | --- | --- | --- | --- |
|  |  |  |  |  |
|  |  | EVT+IVT group (n=486) | EVT-IVT (n=395) | p-value |
| Adverse events | |  |  |  |
|  | Groin hematoma (n, %) | 2 (0.4%) | 3 (0.8%) | 0.662 |
|  | Groin pseudoaneurysm (n, %) | 2 (0.4%) | 2 (0.5%) | 1.000 |
|  | Space occupying edema of MCA territory (n, %) | 18 (3.7%) | 16 (4.1%) | 0.861 |
|  | Intracerebral hemorrhage (n, %) | 20 (4.1%) | 15 (3.8%) | 0.864 |
|  | Myocardial infarction (n, %) | 3 (0.6%) | 4 (1.0%) | 0.707 |
|  | Recurrent stroke (n, %) | 11 (2.3%) | 11 (2.8%) | 0.668 |
| EVT: endovascular therapy, IVT: intravenous thrombolysis, MCA: Medial Cerebral Artery | | | | |
|  |  |  |  |  |
