# Supplementary material for: Inhouse Bridging Thrombolysis Is Associated With Improved Functional Outcome in Patients With Large Vessel Occlusion Stroke: Findings From the German Stroke Registry
Source: Front Neurol. 2021 Jun 10;12:649108. doi: 10.3389/fneur.2021.649108 (PMC8222775; doi:10.3389/fneur.2021.649108)
Supplement: Supplementary file 2 [file Image_2.pdf]

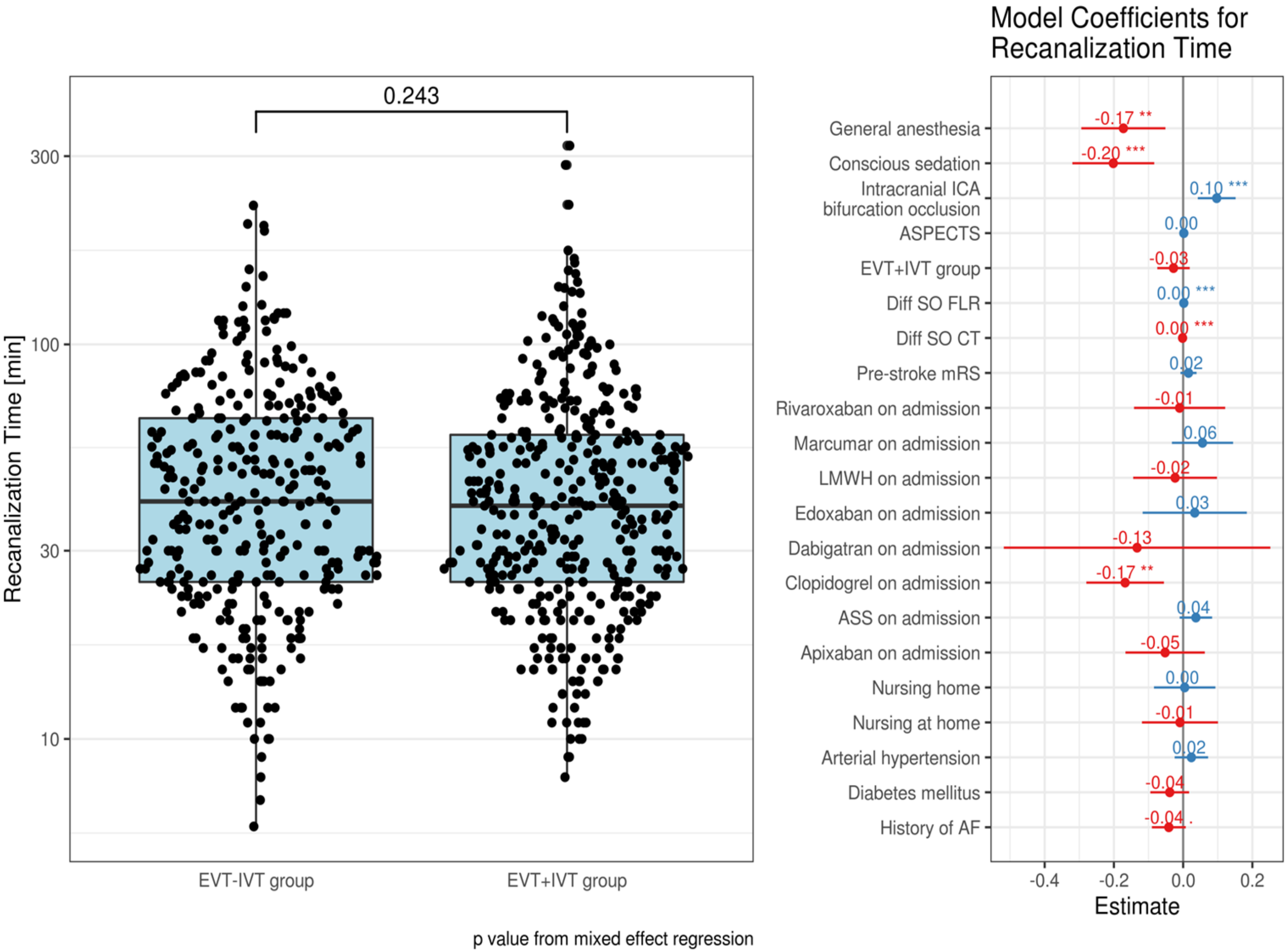

Supplementary Figure 2a: Adjusted analysis of the groin to recanalization times in patients with- (EVT+IVT) and without (EVT-IVT) bridging intravenous thrombolysis (EVT: endovascular treatment, IVT: intravenous thrombolysis, AF: atrial fibrillation, ASPECTS: Alberta stroke program early CT score, ASS: acetylsalicylic acid, LMWH: low molecular weight heparin, mRS: modified Rankin scale, ICA: internal carotid artery).

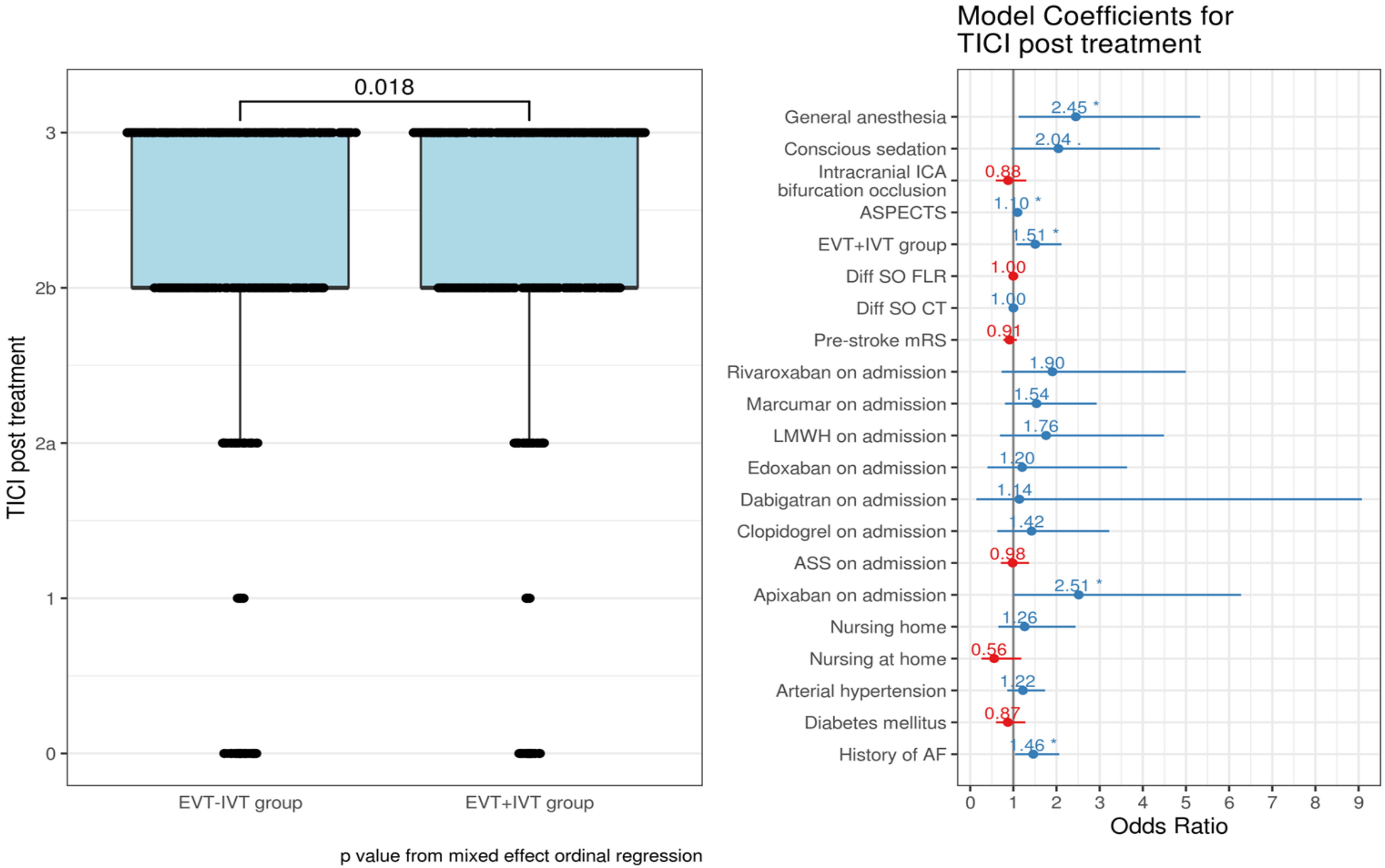

Supplementary Figure 2b: Adjusted analysis of the reperfusion status (quantified by the Thrombolysis in Cerebral Infarction (TICI) scale) in patients with- (EVT+IVT) and without (EVT-IVT) bridging intravenous thrombolysis (EVT: endovascular treatment, IVT: intravenous thrombolysis, AF: atrial fibrillation, ASPECTS: Alberta stroke program early CT score, ASS: acetylsalicylic acid, LMWH: low molecular weight heparin, mRS: modified Rankin scale, ICA: internal carotid artery).

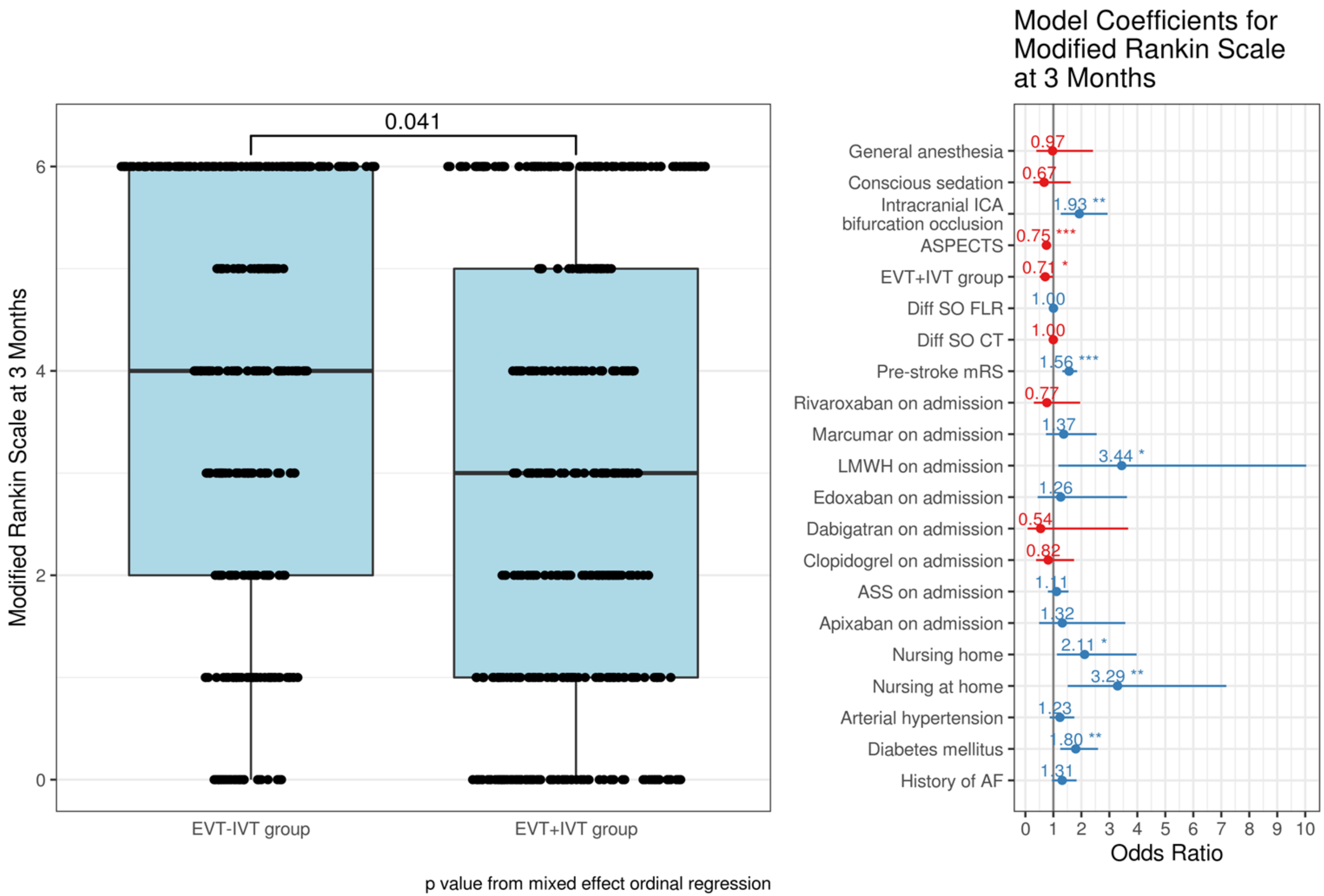

Supplementary Figure 2c: Adjusted analysis of the modified Rankin Scale (mRS) at 90 days in with- (EVT+IVT) and without (EVT-IVT) bridging intravenous thrombolysis (EVT: endovascular treatment, IVT: intravenous thrombolysis, AF: atrial fibrillation, ASPECTS: Alberta stroke program early CT score, ASS: acetylsalicylic acid, LMWH: low molecular weight heparin, ICA: internal carotid artery).

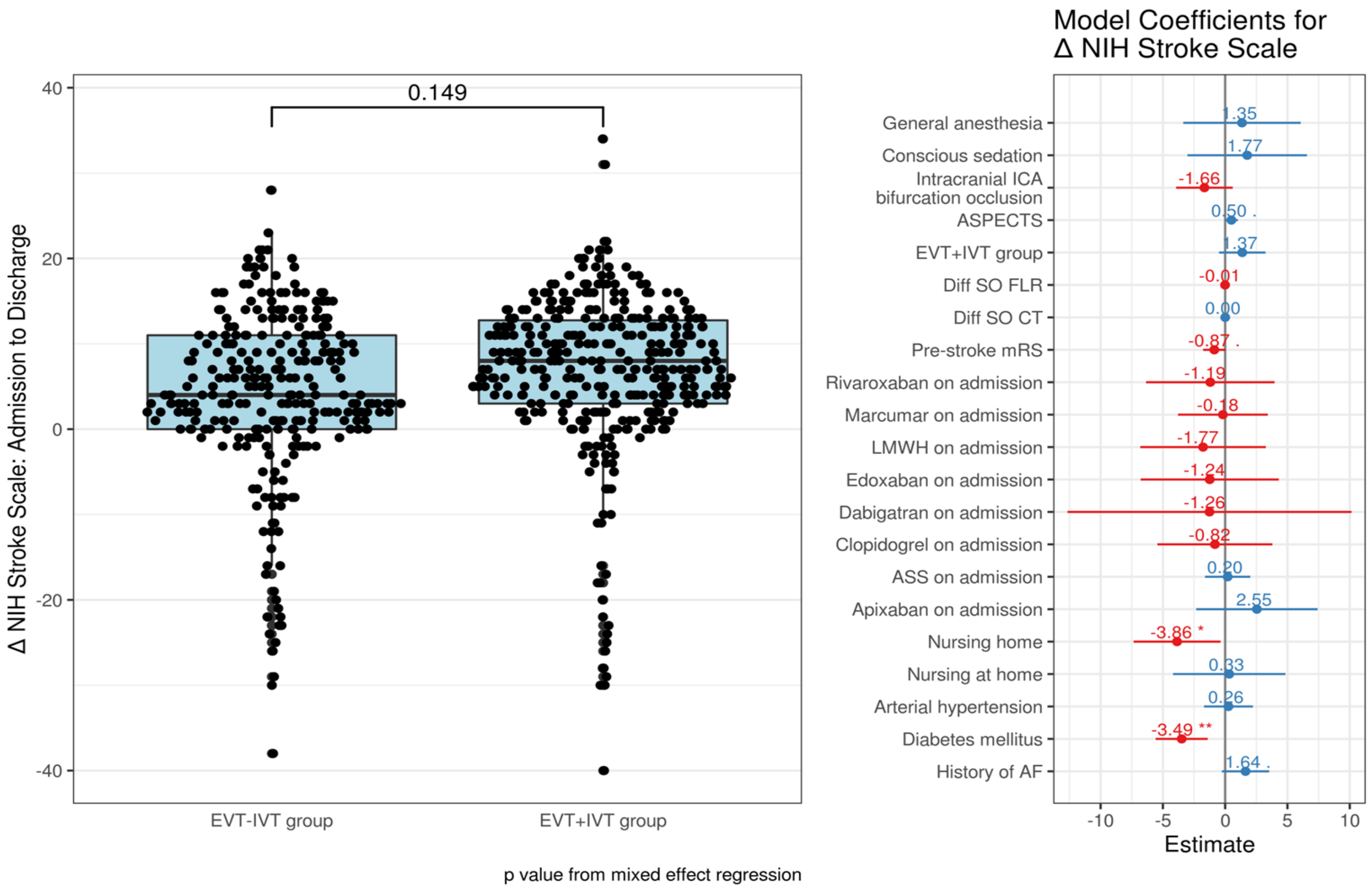

Supplementary Figure 2d: Adjusted analysis of the change in National Institute of Health Stroke Scale (NIHSS) (admission NIHSS minus discharge NIHSS) in patients with- (EVT+IVT) and without (EVT-IVT) bridging intravenous thrombolysis (EVT: endovascular treatment, IVT: intravenous thrombolysis, AF: atrial fibrillation, ASPECTS: Alberta stroke program early CT score, ASS: acetylsalicylic acid, LMWH: low molecular weight heparin, mRS: modified Rankin scale, ICA: internal carotid artery).

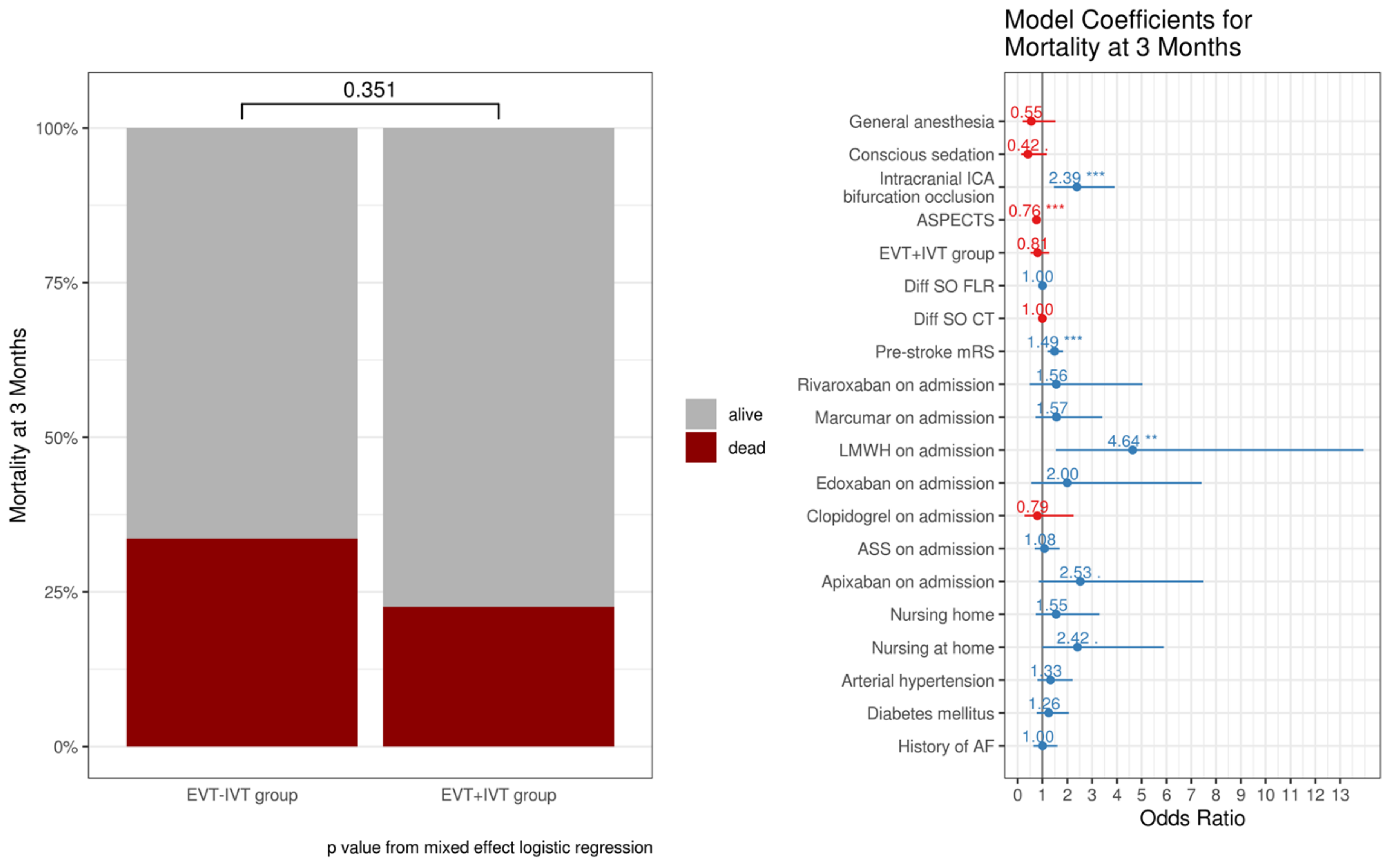

Supplementary Figure 2e: Adjusted mortality analysis at 90 days in patients with- (EVT+IVT) and without (EVT-IVT) bridging intravenous thrombolysis (EVT: endovascular treatment, IVT: intravenous thrombolysis, AF: atrial fibrillation, ASPECTS: Alberta stroke program early CT score, ASS: acetylsalicylic acid, LMWH: low molecular weight heparin, mRS: modified Rankin scale, ICA: internal carotid artery).
