# Supplementary material for: Inhouse Bridging Thrombolysis Is Associated With Improved Functional Outcome in Patients With Large Vessel Occlusion Stroke: Findings From the German Stroke Registry
Source: Front Neurol. 2021 Jun 10;12:649108. doi: 10.3389/fneur.2021.649108 (PMC8222775; doi:10.3389/fneur.2021.649108)
Supplement: Supplementary file 3 [file Image_1.pdf]

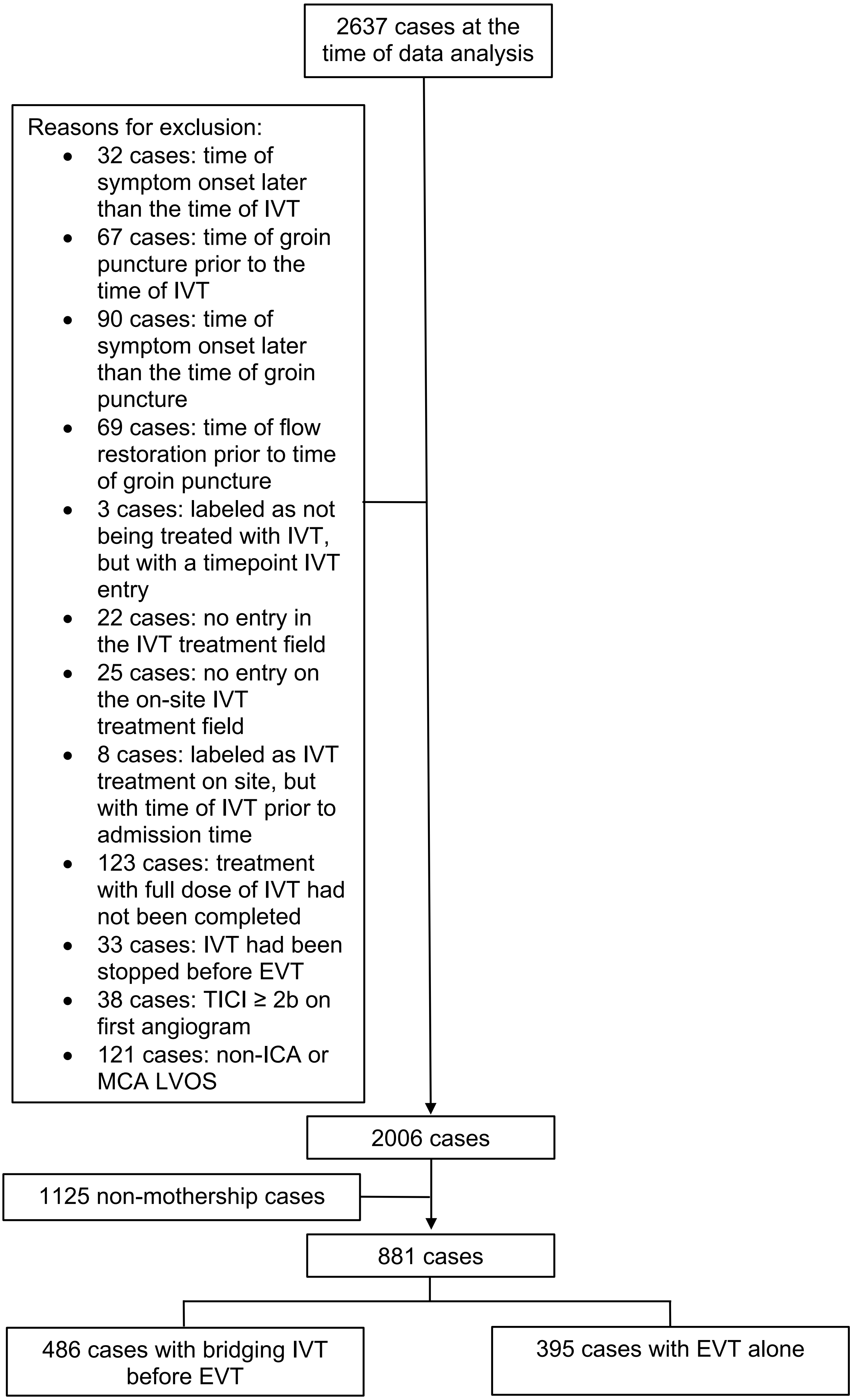
2637 cases at the time of data analysis
Reasons for exclusion:
• 32 cases: time of symptom onset later than the time of IVT
• 67 cases: time of groin puncture prior to the time of IVT
• 90 cases: time of symptom onset later than the time of groin puncture
• 69 cases: time of flow restoration prior to time of groin puncture
• 3 cases: labeled as not being treated with IVT, but with a timepoint IVT entry
• 22 cases: no entry in the IVT treatment field
• 25 cases: no entry on the on-site IVT treatment field
• 8 cases: labeled as IVT treatment on site, but with time of IVT prior to admission time
• 123 cases: treatment with full dose of IVT had not been completed
• 33 cases: IVT had been stopped before EVT
• 38 cases: TICI ≥ 2b on first angiogram
• 121 cases: non-ICA or MCA LVOS
2006 cases
1125 non-mothership cases
881 cases
486 cases with bridging IVT before EVT
395 cases with EVT alone

Supplementary Fig 1: Flow chart showing the selection and reasons for exclusion of patients in the whole German Stroke Registry cohort.
